# Planting a Seed of Experience – Long Term Effects of a Co-curricular Ecogarden-Based Program in Higher Education in Hong Kong

**DOI:** 10.3389/fpsyg.2020.583319

**Published:** 2021-01-15

**Authors:** Chi-Chiu Cheang, Wai-Ki Ng, Yuen-Sam Diana Wong, Wai-Chin Li, Kwok-Ho Tsoi

**Affiliations:** Department of Science and Environmental Studies, The Education University of Hong Kong, *Tai Po*, Hong Kong

**Keywords:** career development, content analysis, environmental awareness, garden-based learning, metacognition, personal growth, phenomenographic study, serial time point sampling

## Abstract

This paper reports on the long-term effectiveness of a non-formal co-curricular educational program based on a campus ecogarden at a Hong Kong university in developing pro-sustainability awareness, attitudes and behavior among undergraduate students. This service-based, nature-based experiential learning program, termed the Ecogarden Farmer and Biodiversity Surveyor, has been running at the university since 2015. The program is divided into two consecutive phases: a training phase comprising various learning activities and a successive internship phase consisting of the all-round practical tasks involved in managing the garden. A retrospective evaluation of the program using phenomenographic approach and content analysis was adopted to reveal the diversity of students’ learning experience, as the indicators of the success of the program. Of 112 participants from 4 cohorts, 32 completed online questionnaires, and semi-structured interviews were successfully conducted with twelve participants, three from each of the four cohorts. The results indicated that the program’s outcomes could be categorized into five themes. Most outcomes fit into the theme ‘an increase in knowledge and skill level,’ followed by ‘rise in environmental awareness,’‘facilitation of personal growth,’ and ‘enhancement of career development.’ Many structural experiences revealed may suggest the success of the program. The longer the participants had participated in the program, the more in-depth and diversified reflection of the senior participants relating to personal development were mentioned. This study provides critical insights into the validity of retrospective program evaluation for assessing the long-term impact of EfS programs by introducing a cross-sectional study of different cohorts as a serial time-point sampling strategy.

## Introduction

Higher education institutions (HEIs) play a critical role in education for a just and sustainable future (Cortese, 2003; Chalkley, 2006; Barth, 2014). Non-formal education, as well as informal learning experience, has been increasingly emphasized for its importance in nurturing students’ competency in and positive attitude toward sustainability within higher education (Tang et al., 2017; Gramatakos and Lavau, 2019). Various co-curricular approaches, such as campus organic learning gardens (Duram and Williams, 2015; Luetz and Beaumont, 2019; Pérez-López et al., 2020), campus-based ecotourism (Robbins et al., 2019), the co-planning of a roof-top garden and wastewater treatment facility by students and university colleagues (Mateus et al., 2020), a marine conservation program (Cheang et al., 2017) and a waste recycling program in campus halls of residence (Cheung et al., 2018), have been found to motivate pro-sustainability behavioral and attitudinal changes amongst undergraduates.

### An Integrated Approach to Cognitive, Psychomotor and Affective Domains of Education for Sustainability

As Shephard (2008) stated, a focus on the cognitive domain of education for sustainability (EfS) has in the past been unsuccessful in HEIs; success in EfS focuses on the affective domain to bring about affective and attitudinal changes. What sustainability attributes should a student have acquired upon graduating from an HEI? Wiek et al. (2011) proposed five core competencies that a sustainability-literate graduate from higher education should possess: systems thinking, anticipatory, normative, strategic and interpersonal competencies. Twelve competencies for sustainability (including those proposed by Wiek et al., 2011) were further summarized by Lozano et al. (2017), a few of which are critical thinking, interpersonal relation, and justice and ethics.

Indeed, innovative teaching methods are needed to facilitate students’ learning and experience of concepts of sustainability and to foster a pro-sustainability attitude amongst them (Ely, 2018). In recent years, ever more pedagogies have proved effective in arousing students’ environmental awareness and nurturing their environmentally conscious attitudes, such as problem-based and project-based learning (Cörvers et al., 2016; Leal Filho et al., 2016), nature-based and/or outdoor experiential learning (Lugg, 2007; Krasny and Delia, 2015), place-based education (Flanagan et al., 2019) and service-based learning (Zint et al., 2014). Other representative pedagogies, such as the jigsaw approach to collaborative learning and mind mapping, were also summarized by Lozano et al. (2017), with corresponding competencies reviewed.

### Garden-Based Learning (GBL)

Planting a garden was cited by Sipos et al. (2008) as one of the best ways to comprehensively engage students’ hands (psychomotor domain), heads (cognitive domain) and hearts (affective domain) in EfS. GBL has a long history of development in the United States (reviewed by Williams and Dixon, 2013). An increasing number of recent studies have explored GBL at different levels of education, driven by two important imperatives of education, food security and health, as well as the exposure of students to nature (Williams and Dixon, 2013; Williams, 2018).

In higher education, GBL has commonly been adopted in non-formal contexts. This approach is appealing to HEIs, as it is effective in altering students’ attitudes without requiring them to possess prior gardening experience or high environmental motivation (de Young et al., 2016). With teachers’ help, a group of students tackled various real-life problems during the management of a campus garden, which was found to profoundly enhance the students’ environmentally conscious attitude (Gaylie, 2009). In another study, a university food garden not only offered experiential learning benefits for students but also genuinely enhanced the environmental sustainability of the campus by providing a local food source (Duram and Klein, 2015). In terms of occupational training, Pérez-López et al. (2020) demonstrated the important role of GBL in the professional competency of pre-service teachers for early childhood education, through experiential learning in natural settings.

Several characteristics of GBL make it a good model for EfS. The experiential nature of GBL is undoubtedly the most important component of any garden-related EfS program. Student-oriented or student-initiated programs can strengthen students’ problem-solving ability and arouse in them a sense of belonging to their university (Skinner et al., 2012). The real-world experience that GBL offers to students (Brundiers et al., 2010) and the authentic problem solving that occurs in the management of a garden should lead students to regard the learning as meaningful, enhancing their engagement (Skinner et al., 2012). The experience of nature offered by GBL is crucial not only for maintaining mental well-being but also for facilitating the cognitive learning process (Keniger et al., 2013; Collado and Staats, 2016). Ethical consideration with regard to environmental issues, civic engagement in the community and professional competency have also been found to be effectively improved by GBL (Aftandilian and Dart, 2013; Pérez-López et al., 2020).

### Ecogarden and the Co-curricular Program

To further enhance GBL on a university campus in Hong Kong, Cheang et al. (2017) used a technology-enhanced ecogarden as a device for EfS. An ecogarden, or ecological garden, is a garden in which ecological management is practiced. Common strategies of management, which are regarded as nature-based and environmentally friendly, include selecting appropriate vegetation species and avoiding artificial fertilizers or chemical pesticides in planting or treating bodies of water (Ying, 2014). Most recently, ecogarden design has incorporated such concepts of environmental sustainability as the sustainable use of energy and resources and conservation of biodiversity, and even the Chinese philosopher Confucius’ idea on the appreciation of the harmony of nature and humans (Cheang et al., 2017). This kind of garden, providing a nexus of sustainability between generations (longitudinal axis) and among stakeholders (cross-sectional axis), is becoming particularly important for the enhancement of conservation, leisure, spiritual and educational value in a city context (Pudup, 2008; Moskwa and Crilley, 2012).

**FIGURE 1:**
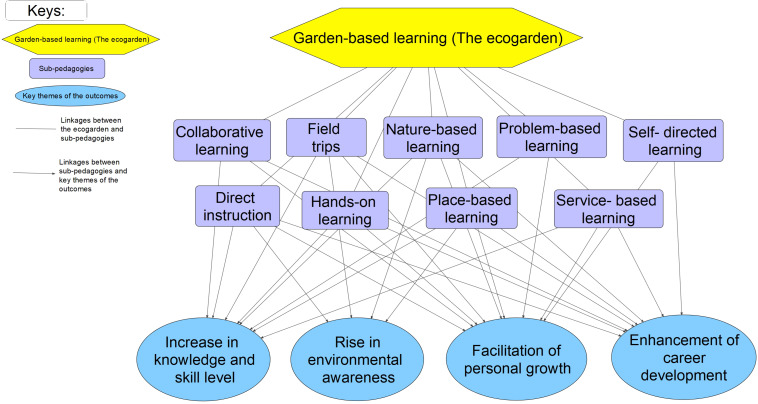
The framework of the ecogarden-based co-curricular program with the garden-based learning as the overarching pedagogy with nine sub-pedagogies. The key themes of outcomes identified in the study were mapped to the corresponding sub-pedagogies. The detail mapping matrix would be referred to Table 1.

To nurture the pro-environmental awareness, attitudes and behavior of undergraduates at a university in Hong Kong with an ecogarden on campus, a non-formal co-curricular educational program was implemented at the university. GBL is the overarching pedagogy of this ongoing program, with nine sub-pedagogies (Table 1 and Figure 1), such as nature-based (Pérez-López et al., 2020), place-based (Flanagan et al., 2019) and service-based sub-pedagogies (Gaylie, 2009; Walter, 2013; Cheang et al., 2017). The training program, called the Ecogarden Farmer and Biodiversity Surveyor, has been in place at the university since 2015, with the objectives of (1) increasing the participants’ (both undergraduates and postgraduates) knowledge of organic farming and biodiversity surveys; (2) arousing students’ interest in and awareness of organic farming, biodiversity conservation and issues of sustainable development; and (3) developing students’ guiding and mentoring skills. This annual program consists of two stages, training and internship. Lectures, field trips and hands-on sessions on various topics, such as an overview of the ecogarden, organic farming, aquaponics, identification and ecological survey of butterflies, birds, plants and herpetofauna, and docent techniques, compose the training stage in the program’s first half. In the internship stage, students assist staff in managing the ecogarden in an all-round manner. Their duties include farming, maintaining the infrastructure of the ecogarden, serving as tour guides/docents to receive local primary and secondary students and teachers, as well as any guests arranged by the university, and designing and implementing an ecogarden-based teaching module for local schools.

**TABLE 1:** The exemplary quotes from the themes identified in the study with the associating sub-pedagogies being matched.

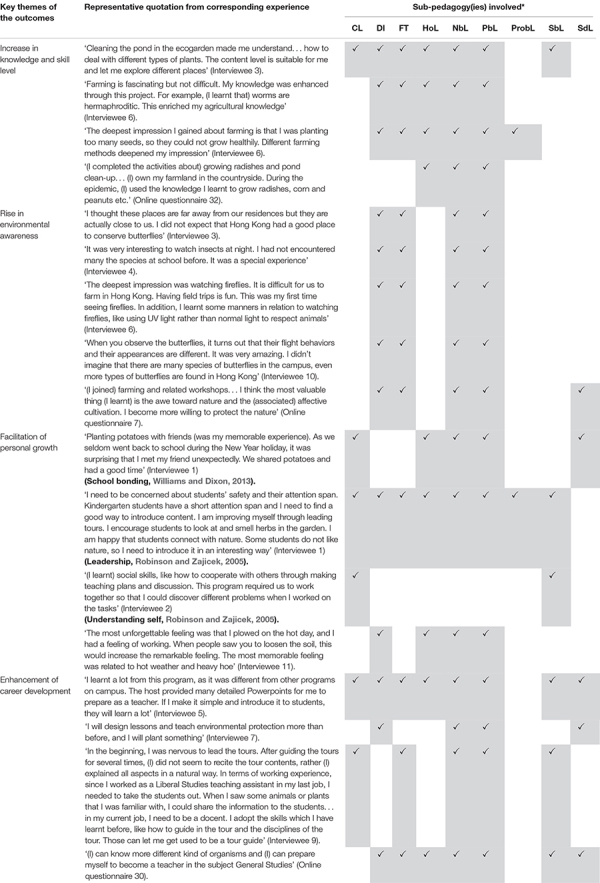

Corresponding psychological constructs documented in previous literatures were identified and bold. * Sub-pedagogies involved in this research were CL, collaborative learning; DI, direct instruction; FT, field trip; HoL, hands-on learning; NbL, nature-based learning; PbL, place- based learning; ProbL, problem-based learning; SbL, service-based learning; and/or SdL, self-directed learning.

### Long-Term Effects of EFS Program

Several studies have focused on how long participants retain the impact of an environmental program (reviewed by Liddicoat and Krasny, 2013), which were mostly outdoor learning activities (e.g., Farmer et al., 2007; Balestri et al., 2017). The longest period of retrospective evaluation research on long-term memories of an environmental program is 45 years (Liddicoat, 2013). Based on these memories, the outdoor learning experience has often been regarded as life-changing for the participants (Kellert, 1998).

Relatively few studies have attempted to reveal the long-term effects of any EfS or environmental education (EE) program at an HEI. A wilderness orientation program conducted at a university left a positive impact on the participants as long as 17 years after their graduation (Gass et al., 2003). Dvorak et al. (2011) found a difference in pro-environmental workplace practices between new interns and alumni joining an intensive course with internships. However, self-perceived environmental ethics played a significant role in governing these practices. Hesselbarth and Schaltegger (2014) examined the long-term effect (over 10 years) of an MBA education program on sustainability management and used the resulting data to construct a competency matrix for the graduates, including subject-specific, methodological, social and personal competencies.

As Liddicoat and Krasny (2013) pointed out in a critique, there has been no consensus on the time frame a retrospective evaluation researcher should use to assess the long-term impact of an EE/EfS program. Even more concerningly, most studies have offered no clear definition of ‘long-term’ impact. Liddicoat and Krasny (2013) used 1 year as a reference point to review studies on long-term impact and found that only a few studies had investigated the impact for longer than 1 year.

Considering the very limited studies on the long-term effects of EfS programs at HEIs, the current cross-sectional study of participants from different cohorts aimed to evaluate the long-term (up to 4 years) effectiveness of an EfS program in nurturing participants’ pro-sustainability knowledge, skills and attitudes, by revealing the experience of the participants and its association with the program. A matrix matching the sub-pedagogies involved in the program with the outcomes observed in the participants was created to serve as a reference for other co-curricular GBL modules of higher education to be implemented in the future.

## Materials and Methods

Being an explorative and evaluative study, this retrospective study adopted a pragmatic worldview, with the phenomenography as the methodological paradigms (Feldon and Tofel-Grehl, 2018). Phenomenographic approach is an empirical approach used in educational research to understand and categorize the “variation in people’s ways of understanding the phenomenon,” which is different from the phenomenological studies that focus on the study of the phenomenon *per se* (Larsson and Holmström, 2007). Phenomenography has its interpretivist ontological root, and yet it also stresses on the existence of a real, objective world (non-dualist ontology; Marton, 2000; Reed, 2006). The learning processes of participants associating with the ecogarden-based program in this study was different from participants to participants, as well as, from learning contexts to contexts (Han and Ellis, 2019). The personal conception is the key for the learner to understand the physical world through personal interpretation of the experience gained in the program (Feldon and Tofel-Grehl, 2018).

Epistemologically, phenomenographic studies in education investigate the purposefulness and consciousness of the students to learn (Han and Ellis, 2019). The experience gained during the learning process may be interpreted differently by the learners. The major aim of phenomenographic study is to understand the diversity of the learning experience the learners gained and how they made sense from them.

To assess the effectiveness of the focal program through method triangulation, we studied the diversity of the learning experience through two data analyzing approaches, the phenomenographic study *sensu stricto* (Marton, 1981) and content analysis (Bogdan and Biklen, 2007). Both approaches, sharing the non-dualistic ontology, adopt a second-order view of the development of knowledge (Trigwell, 2000; Reed, 2006). We tried not to make any statement (the outcome in this study) about the phenomenon directly but through the experiences described by the participants. The only difference between the two approaches is whether the variation of experience reflected by the participants were internally correlated (Reed, 2006).

### Participants

Purposive sampling on a voluntary basis was used to recruit participants from students involved in the focal EfS program. All of the students (112 candidates) enrolled in the program, including both alumni and current students studying diverse educational (e.g., General Studies, Language Education, and Early Childhood Education) and non-educational major subjects (e.g., Global and Environmental Studies etc.) in four cohorts, were invited to take part in an online questionnaire survey. 32 participants completed online questionnaires were received, giving a response rate of 28%. The respondents of online questionnaire were consisted of 9 males and 23 females. To further explore the views of participants from different cohorts, follow-up semi-structured interviews were successfully conducted on twelve of the participants with 3 males and 9 females (three from each cohort), based on their preferences as indicated in the online survey. The majority (66.67%) of the interviewees studied education-related fields, including English Language Education and General Studies etc. The sample size is more than what Dahlgren (1995) and Åkerlind (2005) suggested for phenomenographic study to capture the variation.

The protocol of the study reported was approved by the Human Research Ethics Committee of the Education University of Hong Kong (Ref. No.: 2019-2020-0312). The participants were informed of all relevant information on the study before taking part. They understood their right to withdraw from the study at any time point if they chose not to participate. For the online questionnaire, all of the participants gave their consent to participate by proceeding from a consent statement shown in a webpage before filling out the questionnaire. For the video-conferencing interviews, written consent to take part in the study was obtained.

### Data Collection

Invitations to join the study were sent via the alumni and current students’ e-mail addresses, as registered on their first enrolment in the program. All of the participants were invited to complete an online questionnaire directed at collecting their views on the program. Demographic data, as well as information on self-perceived degree of participation in the program, given on a 5-point Likert scale (where 5 indicated the most active participation), were collected from the respondents. The other questions asked were all open questions aimed at understanding why the participants had joined the program, how the participants reviewed the program and what impact the program had had on the participants (Supplementary Appendix 1).

In addition to the online questionnaire, follow-up semi-structured interviews were conducted on 12 students via online videoconferencing. Each interview lasted for 30 minutes. Any particular view on or impact of the program was further elaborated in these interviews. The interview questions were open-ended and were based on the responses to questions that the students had provided in the online questionnaire. The basic approach in the interview was to ask ‘Why’ and reveal more in-depth latent contents and impact from the students’ perspective. Some example questions are listed in Supplementary Appendix 2.

### Data Analysis

Each interview was video-recorded, and the conversations were all transcribed. The text from both the online questionnaire and the transcription of the interviews was inductively analyzed according to the two data-analyzing approaches described below.

#### Phenomenographic Study

We adopted the phenomenographic method of Säljö (1997) to understand students’ experience and interpretation. Six steps, namely familiarization, identification, sorting, contrasting, categorizing, and reliability checking, were completed to analyze the text from both the online survey and transcript of the interviews.

The process of categorization is iterative in nature. Refinement and modification, if needed, were taken place in order to generate a final set of categories, which would best represent the diversity of the participants’ experience. For the categorization, we follow the three requirements, the distinctness, parsimony and logical relationship of categories, as stated by Han and Ellis (2019). To tally with the analysis of content analysis, we use “theme” for referring to the categories identified in phenomenographic study.

The categorization was conducted by one experienced researcher. A second experienced researcher independently checked the coding and categorization and deliberated with the first researcher in cases of inconsistency. A third researcher was consulted if the inconsistency could not be resolved.

#### Content Analysis

The latent content in our conversations with the interviewees and their responses to the online survey were coded inductively. The codes were regarded as the experience related to the long-term program outcomes. They were reviewed, decontextualised, categorized, and reorganized into themes to explore any hidden or shared points of view and/or structures of the interrelated codes among different interviewees (Bogdan and Biklen, 2007). Eventually, a matrix of the sub-pedagogies involved and their corresponding outcomes was constructed based on the themes categorized. Similar categorization procedure to phenomenographic approach by three researchers was adopted as quality control measures.

As for content analysis, the occurrences of the codes (outcomes) and themes were analyzed using the computer software NVivo 11 Pro. To elucidate the outcomes and thus the effectiveness of the program, the relationships among the codes and themes were referred to the documented psychological constructs.

The data from the content analysis were shown in the form of descriptive statistics, with graphs constructed using the software GraphPad Prism 8. Maps showing the psychological constructs described by the participants were generated using VUE Version 3.3.0. The corresponding experiences of/pedagogies experienced by the participants in the program were also mapped on to those psychological constructs to synthesize a general model of the pedagogical devices used in the program and their respective outcomes.

## Results

### Major Themes Identified From Both Phenomenographic Approach and Content Analysis

Four themes of outcomes could be categorized from both the questionnaires and the semi-structured interviews: ‘increase in knowledge and skill level,’ ‘rise in environmental awareness,’ ‘facilitation of personal growth,’ and ‘enhancement of career development,’ with miscellaneous outcomes being classified as ‘others,’ as revealed from both phenomenographic studies and content analysis. Based on the content analysis, on average, two to three codes per participant were mentioned by the respondents to the online survey. The codes recorded in the online survey were generally simple and superficial, without much elaboration and example illustration. The outcomes collected from the interviewees ranged from 8 to more than 20 codes per participant. The experienced shared by the interviewees appeared more in-depth and reflective, with more supporting examples.

**FIGURE 2:**
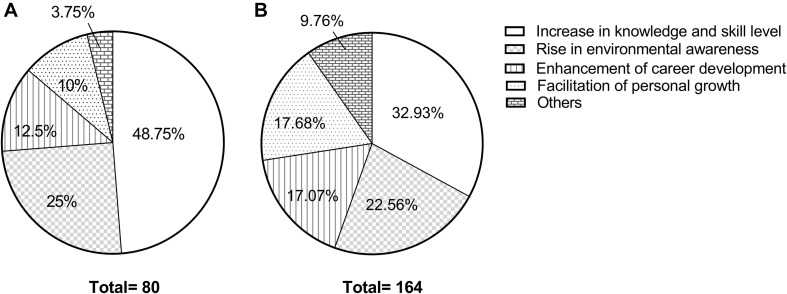
Frequency of the outcomes mentioned by the respondents in **(A)** the online questionnaire and **(B)** video-conferencing interviews.

Albeit with the slight actual percentages difference between the online survey and videoconferencing interviews, the theme ‘increase in knowledge and skill level’ is the theme with the most codes, followed by the theme ‘rise in environmental awareness,’ ‘enhancement of career development,’ and ‘facilitation of personal growth’ (Figure 2).

#### Increase in Knowledge and Skill Level

**FIGURE 3:**
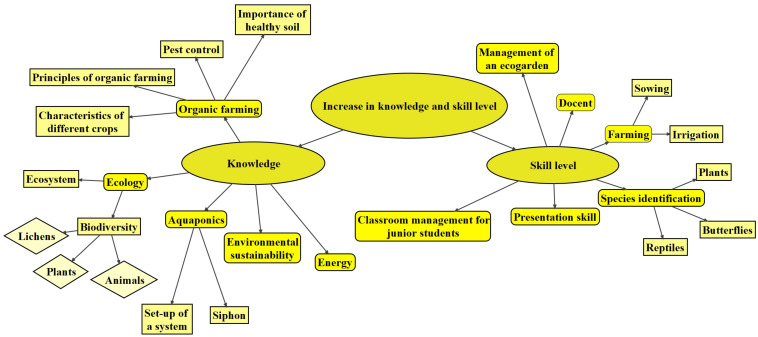
Mapping of the codes under the theme ‘Increase in knowledge and skill level.’ Different shapes refer to different levels of themes.

Within the theme of ‘increase in knowledge and skill level,’ the codes that could be recalled by the participants were essentially aligned with what had been taught in the training workshops. The subthemes identified included the knowledge on organic farming, aquaponics, ecology, environmental sustainability and energy, as well as, the skills on ecogarden management, farming, species identification, docent, presentation and classroom management for junior students (Figure 3).

Most of the knowledge that the participants described was examples of system knowledge (Frick et al., 2004), such as knowledge of biodiversity, principles of organic farming and characteristics of different crops; and action-related knowledge, consisting of skills such as those required for species identification and presentation in front of a crowd. For example, Interviewee 6 stated that “Farming is fascinating but not difficult. My knowledge was enhanced through this project. For example, (I learnt that) worms are hermaphroditic. This enriched my agricultural knowledge… I learnt preparation and presentation skills through this program.” Interviewee 11 talked about her farming experience and reflected about the biodiversity she encountered, “When I plow, I can feel that lives and insects are found in the soil. Normally, you won’t pay attention on insects or even when you loosen the soil deliberately. Once you plant the crops, you will see other (hidden) things (living organisms) inside.”

There were some examples of effectiveness knowledge, which were related to the facilitation of sustainability decision making, such as the importance of healthy soil, pest control and aquaponics. Interviewee 6 mentioned that “The practical skill (that I learnt) is the operation of an aquaponics equipment. I needed to consider water and other hardware. The ratio of fish to vegetables is important. For farming, I saw (learnt about) many tools, such as pots, hoes, and pesticides, and considered the weather (for farming) as well.” Systems-thinking competence was also reflected in the interviews. The response of Interviewee 10 stated that “(I learnt about) The relationship between the ecology of the insects, butterflies and plants in Hong Kong, their overall distribution in Hong Kong, the concept of farming, and the aquaponic system. I think it’s very comprehensive.”

#### Rise in Environmental Awareness

**FIGURE 4:**
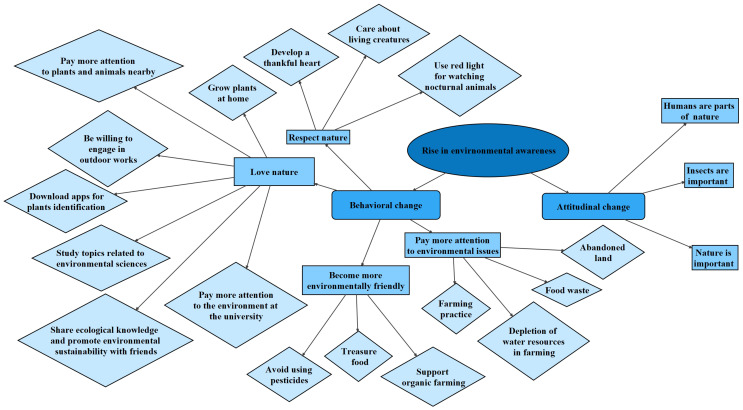
Mapping of the codes within the theme ‘Rise in environmental awareness.’ Different shapes refer to different levels of themes.

Within the environmental themes, the codes could be broadly classified into two subthemes attitudinal and behavioral changes (Figure 4), which were related to the realization of students on the two elements of the collective importance of the environment (Flanagan et al., 2019), ‘the natural resources on which life depends’ and ‘collective actions to protect a community’s resources.’

On the attitudinal change, many participants have reflected the latent content about environmental empathy and attitude toward gardening (Williams and Dixon, 2013), which was expressed as nature loving and respecting (Figure 4). Some example quotes were “I have a thankful heart now, as different types of crops are not easy to grow. I eat different types of crops with gratitude now.” (Interviewee 6), “It was practical for me to enhance my learning through farming. This also made me understand the importance of sustainability and that I am part of the nature.” (Interviewee 7), “I fell in love with flowers and plants.” (Online survey) and “Organic farming is about eating crops in the right season… Understanding the scenarios involved in growing vegetables in China, such as the shortage of water in Yin Chuan, I thought further about sources of food and ways of farming.” (Interviewee 1). Some of these attitudinal changes could be associated with the higher level and more in-depth reflections such as those on the self-efficacy (Sherer et al., 1982). Interviewee 1 expressed a deep reflection on her current living style and was eager to make a change, “Farmers live and work on farms without switching on air conditioners. I think that this kind of lifestyle is good. I am willing to try this simple life by living on the farm. I can try to have a lifestyle of “half farming, half working.”

On the behavioral change, actual change on knowledge enhancement such as “I have downloaded an app to understand different types of plants.” (Interviewee 5) and change to a more environmentally friendly behavior for instance “I tried to plant more. See whether I can create a similar program, as my home is adjacent to a farm.… We can also collect food waste in the school, and I have more awareness on it.” (Interviewee 7) were commonly recorded in the interviews. Some of the behaviors reported were not just restricted to the self-competence for environmental protection, but also on the influence to the surrounding people, “I can share my experience with others to promote organic farming and sustainable development.” (Online survey).

It is worth-noting that many of the outcomes recorded, such as ‘develop a thankful heart,’ ‘being willing to work outdoors,’ ‘share ecological knowledge with friends,’ and ‘treasure food’ (Figure 4), were neither explicitly taught nor mentioned in the training of the program.

#### Facilitation of Personal Growth

**FIGURE 5:**
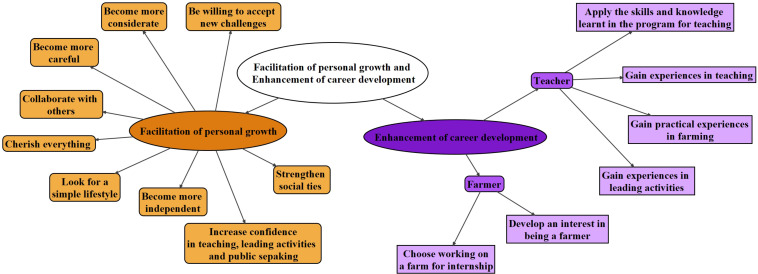
Mapping of the codes under the themes ‘Facilitation of personal growth’ and ‘Enhancement of career development.’ Different shapes refer to different levels of themes.

The content of the outcomes under the themes ‘Facilitation of personal growth’ varied from subthemes of becoming more considerate, independent and confident, to the subthemes on the willingness to strengthen students’ own social ties, accepting new challenges and even the attempt to live a simple lifestyle (Figure 5).

For instances, Interviewee 2 gained a significant pleasant experience in teaching an ecogarden-based lesson which boosted up his self-confidence. “This was the first time I had taught a class of students. I had a bad experience when leading a tour in the Wetland Park (in the past). This time I did better and inspired my students to learn more. This increased my self-confidence as I had seldom talked in front of a large number of people before… I will not be shy anymore in the future, or in workplace, as well as other activities. I can have more ways to develop myself.”

Becoming more open to accept new challenges were recorded in both the online survey and interviews, for example, “I became more likely to think and practice when facing new things.” (Online survey), “I have been more confident to step out of my comfort zone (after participating the program).” (Interviewee 11).

New perspectives have been reflected by and introduced to the participants. A significant perceptual change on the interviewee’s ability to solve problem has been documented. Interviewee 1 described her experience as below “Using planting as an example, sometimes we think this crop cannot be grown, but actually it can grow very fast. We face different difficulties and need to try our best to face (and address) them.”

All of the codes recorded under the theme of personal development were not explicitly taught or mentioned in the courses during the training stage of the program. Those were instead the outcomes that the students experienced implicitly from the program or generated upon reflection after graduating from the university.

#### Enhancement of Career Development

Similar to the theme on personal growth, the content of the outcomes under the themes ‘Enhancement of career development’ (Figure 5) was not mentioned in the courses during the training stage of the program. For career development, two major subthemes, being teacher and farmer, were identified.

The codes under the subtheme ‘being teacher’ were commonly found in the participants, especially for those who were studying education major. “I will introduce myself (for having farming experience) when I obtain opportunities in teaching… Plant-related topic are usually covered in the curriculum of the primary school. I can inspire kids through this (ecogarden-related) knowledge if it fits the teaching plan. If not, I will show the content as supplementary knowledge.” Interviewee 5 reflected. She also made in-depth reflection about how the experience gained from the program facilitated her teaching: “I need to have more preparation as children would ask me many extra questions which are out of the books (during the tours).” Another online reply also mentioned that “Education in mainland China does not pay much attention to environmental education. So, (this experience) helped me (as a foreign student coming from mainland China) to prepare for teaching by using eco-garden as a tool (when I come back to China to teach).”

On the subtheme ‘being farmer’, the main impacts of the program were to change the perception of the participants on the agriculture of Hong Kong and to equip the students with the necessary skills in farming. Interviewee 1 stated that “This (experience) affected my choice of firm for my placement. I wanted to further explore farming or eco-tourism.”

#### Others

Other themes such as mental health, sense of belonging to the universities were recorded. Volunteerism (Robinson and Zajicek, 2005) was reflected by some participants as well, together with a strong sense of attachment to the university. Interviewee 3 stated that “I am proud of myself for contributing to my university by serving at the pond (in the ecogarden)… My sense of belonging to the university was increased.” Online replies categorized under this category include “I always discuss knowledge of different organisms with the (ecogarden) staff. We learnt from each other and encouraged each other.”, “I am happy to see that radishes have grown up” and “I was surprised by the growth of plant every time when I water the plant.”

Codes about improving mental health were encountered more than once. Interviewee 1 mentioned about the function of planning in relieving her negative feeling. Data from the online survey also highlighted that “Irrigating plants in the eco-garden can help relieve my anxiety and depression.” Apart from getting rid of negative emotion, Interviewee 11 regarded the farming experience a very happy one. “I loosened the soil at university on a very hot day. When people passing by there, I was a bit embarrassed, and yet happy. The reason is that I like to do these things (farming)… And I will continue to do so.” There was one interesting finding from the interviews. Interviewee 5 particularly pointed out that “I have more interaction with parents about planting.” due to the development of common hobby in their leisure time with her parents.

### Association Between the Themes of Learning Outcome Achieved and the Sub-Pedagogies Revealed by Content Analysis

All of the nine sub-pedagogies were found to be related to the outcomes reported by the participants (Table 1 and Figure 1).

Direct instruction, hands-on learning, nature-based learning and place-based learning were the sub-pedagogies most commonly associated with an increase in knowledge and skills (Table 1). For example, the hands-on experiences in cleaning the pond in ecogarden render Interviewee 3 familiarize the types of plants: “Cleaning the pond in the ecogarden made me understand… how to deal with different types of plants. The content level is suitable for me and let me explore different places,” Interviewee 6 recalled an impression of knowing “less is more:” “My deepest impression I gained about farming is that I was planting too many seeds, so they could not grow healthily. Different farming methods deepened my impression.”

Field trips, hands-on learning, nature-based learning and place-based learning were more closely associated with the change in environmental awareness or attitude theme (Table 1). Interviewee 10 mentioned that “When you observe the butterflies, it turns out that their flight behaviors and their appearances are different. It was very amazing. I didn’t imagine that there are many species of butterflies in the campus, even more types of butterflies are found in Hong Kong.” From the experience of a field visit to know about fireflies at night, Interviewee 4 thought that “It was a special experience” since he “had not encountered the species at the university before.”

For personal growth, collaborative learning was the sub-pedagogy related to most of the representative experiences, followed by hands-on learning, nature-based learning, place-based learning and service-based learning (Table 1). Interviewee 2 reflected that “(I learnt) social skills, like how to cooperate with others through making teaching plans and discussion. This program required us to work together so that I could discover different problems when I worked on the tasks.” Throughout the docent service offered by Interviewee 1, she realized that she needed to “concern about students’ safety and their attention span.” She reflected that “Kindergarten students have a short attention span and I need to find a good way to introduce content. I am improving myself through leading tours. I encourage students to look at and smell herbs in the garden. I am happy that students connect with nature. Some students do not like nature, so I need to introduce it in an interesting way.”

On the career development, students gained authentic experience through direct instruction, nature-based, service-based and self-directed learning (Table 1). Interviewee 7 stressed the value of the experience in design ecogarden-based lessons: “I will design lessons and teach environmental protection more than before, and I will plant something.” Interviewee 9 demonstrated a very in-depth reflection on how the ecogarden-based experience help her in her career development: “In the beginning, I was nervous to lead the tours. After guiding the tours for several times, (I) did not need to recite the tour contents, and rather (I) explained all aspects in a natural way. In terms of working experience, since I worked as a Liberal Studies teaching assistant in my last job, I needed to take the students out to the field. When I saw some animals or plants that I was familiar with, I could share the information to my students… In my current job, I serve as a docent. I adopt the skills which I have learnt before, like how to guide in the tour and manage the disciplines of the tour. Those can let me get used to be a tour guide.”

In particular, place-based and service-based learning pedagogies, with the recognition of the symbolic role of the ecogarden for the university, seemed to nurture in the students a sense of belonging to the university. As shown before, Interviewee 3 stressed that “I am proud of myself for contributing to my university by serving at the pond (in the ecogarden)… My sense of belonging to the university was increased.”

#### Factors Affecting the Learning Outcome Achieved- the Cohort of Enrolment

**FIGURE 6:**
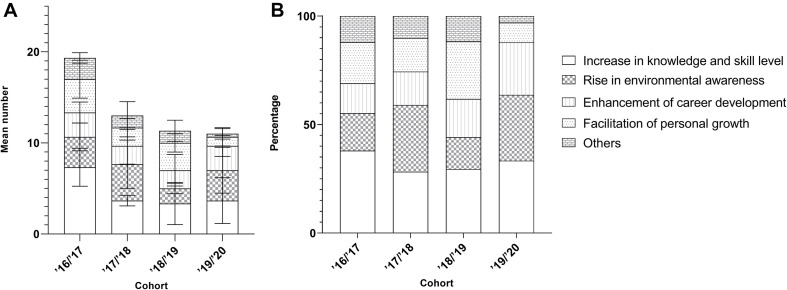
Mean number **(A)** and the corresponding percentage **(B)** of outcomes (±SD) mentioned by the interviewees from different cohorts.

**FIGURE 7:**
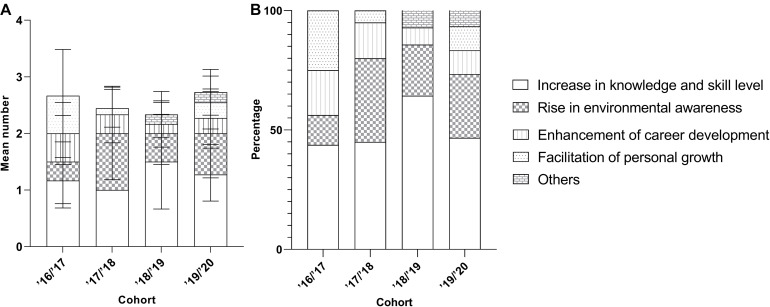
Mean number **(A)** and the corresponding percentage **(B)** of outcomes (±SD) mentioned by the respondents to the online questionnaire from the four different cohorts **(A)** and their composition.

Examining the relationship between the learning experience (outcomes) under different themes and the cohort of enrolment based on content analysis, we can observe that more codes were recorded for the senior cohorts from the interview dataset (Figure 6A), but not for online survey (Figure 7A). While there was no obvious cohort-specific trend in terms of both average number and percentages of codes under different themes (Figures 6, 7), there was more average number and percentages of codes under theme ‘Facilitation of personal growth’ from the senior cohorts as revealed from the online survey (Figure 7).

Phenomenographically speaking, reflections on personal growth coming from the senior cohort during interview were, indeed, more in-depth and diversified. For instance, Interviewee 1 coming from cohort of ‘16/’17 did not just discuss about the local agriculture, but also extended her awareness on the situation of agriculture in other area. “Organic farming is about eating crops in the right season… Understanding the scenarios involved in growing vegetables in China, such as the shortage of water in Yin Chuan, I thought further about sources of food and ways of farming.” She is also the one who expressed a deep reflection on her current living style and was eager to make a change, “Farmers live and work on farms without switching on air conditioners. I think that this kind of lifestyle is good. I am willing to try this simple life by living on the farm. I can try to have a lifestyle of “half farming, half working”.” Interviewee 2 from the same cohort could explain clearly how the experience gained from the program changed himself: “This was the first time I had taught a class of students. I had a bad experience when leading a tour in the Wetland Park (in the past). This time I did better and inspired my students to learn more. This increased my self-confidence as I had seldom talked in front of a large number of people before… I will not be shy anymore in the future, or in workplace, as well as other activities. I can have more ways to develop myself.” Interviewee 2 is also the one who ascertained the role of corporative learning in enhancing his social skill with clear justifications: “(I learnt) social skills, like how to cooperate with others through making teaching plans and discussion. This program required us to work together so that I could discover different problems when I worked on the tasks.”

In contrast, the participants from junior cohort focused on more practical issues and environmental issues. For example, Interviewee 6 from cohort ‘18/’19 remembered the practical skill he learnt about aquaponics: “The practical skill (that I learnt) is the operation of an aquaponics equipment. I needed to consider water and other hardware. The ratio of fish to vegetables is important. For farming, I saw (learnt about) many tools, such as pots, hoes, and pesticides, and considered the weather (for farming) as well.” Interviewee 5 from the same cohort have downloaded an app to understand different types of plants.

#### Factors Affecting the Learning Outcome Achieved- the Self-Perceived Participation

**FIGURE 8:**
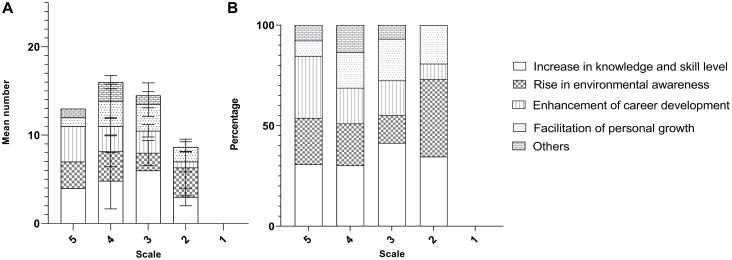
Mean number **(A)** and the corresponding percentage **(B)** of outcomes (±SD) mentioned by the interviewees from different levels of self-perceived participation.

**FIGURE 9:**
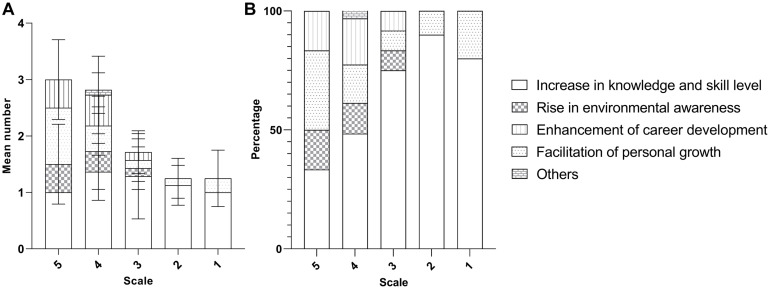
Mean number **(A)** and the corresponding percentage **(B)** of outcomes (±SD) mentioned by the respondents to the online questionnaire from the different levels of self-perceived participation.

The students rated themselves to have deep engagement with the program did not demonstrate a higher number of recorded codes in both interview (Figure 8A) and online survey (Figure 9A). In terms of the composition of the codes under different themes, no participation-specific trend for most of the themes was recorded, except the improvement for career development (Figures 8B, 9B). There is a higher number of codes under career development from the respondents with higher self-perceived participation in the program.

In fact, Interviewee 5 who discussed in-depth how the program experience helped with her teaching duty has rated herself 4 out of the 5 Likert scale in terms of self-perceived participation: “I will introduce myself (for having farming experience) when I obtain opportunities in teaching… Plant-related topic are usually covered in the curriculum of the primary school. I can inspire kids through this (ecogarden-related) knowledge if it fits the teaching plan. If not, I will show the content as supplementary knowledge.” In contrast, Interviewee 6, who rated himself 2 out of 5 in participation, did not mention much on career development, despite of the fairly reflective content was recorded from him, e.g., “I have a thankful heart now, as different types of crops are not easy to grow. I eat different types of crops with gratitude now,” My deepest impression I gained about farming is that I was planting too many seeds, so they could not grow healthily. Different farming methods deepened my impression etc.

## Discussion

### “Dissecting” the Experience Documented in the Study

From the phenomenographic perspective, the experience gained in the learning process could be “dissected” into referential and structural aspects (Marton and Pong, 2005). Han and Ellis (2019) defined the two aspects of experience as “While the former (referential aspect) refers to the meaning of an experience, the latter (structural aspects) is related to the structure of that experience.” For the structural aspects, it could be further classified as the external horizon (discernment of the whole from the context) and internal horizon (discernment of the parts and their relationships within the whole) (Han and Ellis, 2019).

Both referential and structural aspects of experience could be identified in all the themes, and yet many of the online data seemed to be the referential ones, such as “(I learnt about) the campus’s ecology and biodiversity.” As shown by the dataset in the results, many structural aspects of experience were reflected by the interviewees. For instance, Interviewee 5 described how the experience in organizing teaching activity in ecogarden strengthened her competency in organizing similar activity in local school (external horizon of structural experience), Interviewee 1 extrapolated what she learnt about farming and local agriculture to comment on the agriculture oversea (external horizon of structural experience), Interviewee 6 recalled the requirements and conditions for aquaponic farming (internal horizon of structural experience) etc.

The participants’ in-depth reflections exhibiting various structural experience have provided evidence to prove the success of the program. The experience under the themes, ‘Facilitation of personal growth’ and ‘Enhancement of career development,’ which were not explicitly mentioned or taught in the workshop, in fact, hints on the possibility that factors other than curriculum, such as the diverse sub-pedagogies applied, the field-based and authentic service-based working environment etc., could facilitate the gaining of structural experience. The probability for the students to apply the learning experience in other personal context, such as Interviewee 5 practicing planting with her parents and applying her teaching experience in her own working school, would enhance the structural experience being extrapolated to external horizon.

### Evaluating the Program From a GBL Perspective

The curriculum developed in the current co-curricular program relied heavily on scientific, more specifically biological, content. This is in line with the review of Williams and Dixon (2013), suggesting that science and mathematics were the curriculums most relevant to and frequently connected with GBL in primary and secondary schools. In terms of topics covered, the curriculum of our program shared most of the common topics taught for GBL, such as soil chemistry, plant taxonomy, seed germination and its variability, insects (butterflies in our program) and other wildlife (herpetofauna and birds in our program), ecology and environmental horticulture, insects and diseases in farming (Williams and Dixon, 2013).

A distinctive characteristic of the current program is the inclusion of training in presentation and docent skills, which has relatively rarely been mentioned in the literature. The usefulness of the knowledge and skills learnt by the participants to their career development was frequently acknowledged in the dataset. The success of this part of the training can be attributed to the abundance of opportunities for the participants to guide authentic tours during the practical internship stage.

Williams and Dixon (2013) argued that academic performance should be included as part of the assessment to evaluate the effectiveness of GBL. Our program includes no such academic assessment for the co-curricular program, but does provide an award to recognize the effort (service hours) expended by the participants after the completion of the program in 1 year. We suggest that at HEIs, the ‘soft’ skills learnt from the ecogarden experience would be more important than systematic knowledge in benefiting the graduates in the future. The absence of assessment and the inherently non-formal nature of the program’s setting enhanced the students’ motivation to learn. Indeed, one student commented that “students can learn freely (in the program).” Another participants downloaded a plant identification mobile app introduced in the workshop and used it to identify the plants encountered every day.

Compared with other studies adopting GBL, we found that the GBL approach induced all four types of outcome (Williams and Dixon, 2013): (1) personal, social, physical and moral development for self-concept, self-esteem and motivation; (2) pro-environmental attitude and empathy; (3) strengthened food literacy; and (4) ‘school bonding, parental involvement and formation of community’ (Williams and Dixon, 2013). Unsurprisingly, the majority of codes came from scientific knowledge and skills, as the curriculum of the program was most closely connected to science.

Apart from cognitive outcomes, we were able to identify affective responses from the participants, in particular from the interview data. We found evidence from the participants regarding the self-determination model of motivation (Skinner et al., 2012). The students realized their own autonomy, competence and intrinsic motivation, which facilitated their participation in garden management and farming practices (Skinner et al., 2012), for example “I am proud of myself for contributing to my university by serving at the pond (in the ecogarden).”

### Evaluating the Program From EfS Perspectives

Regarding the literature gaging the long-term impact of an EfS/EE program by retrospective assessment, the outcomes revealed by the current study were found to be comparable to psychological constructs or competencies found in other studies. We recorded experiences, such as ‘Increased social interactions,’ ‘Opportunities for personal growth,’ ‘More positive environmental attitudes,’ ‘Self-confidence,’ that have also been reported in previous studies (Liddicoat and Krasny, 2013). For instance, the participants demonstrated a similar understanding and appreciation of nature, which facilitate behaviors associated with conservation and outdoor recreation activities (Liddicoat and Krasny, 2014).

Focusing on HEIs, we identified competencies that resemble those elucidated by Hesselbarth and Schaltegger (2014). The knowledge-and-skills-related constructs identified in the current study could be recognized as the subject and methodological competencies identified in the graduates in their project. The personal growth theme in our study corresponds to the personal competencies identified by Hesselbarth and Schaltegger (2014). However, our study did not reveal the theme of ‘challenging assumptions of self and others,’ as shown in the study of Gass et al. (2003).

### Long-Term Effect of the Program

Our results not only echoed the findings of previous studies demonstrating the long-term effect of the EfS program, but also revealed a likely time-dependent increase in the codes under the personal growth theme after the completion of the program. Besides, the participants who did rate themselves as actively involved in the program reflected more content on the occupational theme. Indeed, none of the codes recorded in the personal growth and occupational themes, were found to be explicitly taught in the program.

The more in-depth and diversified reflection of the senior participants implies that the participants had ‘discovered’ or ‘evaluated’ the outcomes of the program independently after joining the workforce in the community. It could be a results of a self-reflection on the part of the participants and metacognitive reflection on the experience that they had accumulated in the co-curricular program. The findings of this study suggested that, rather than taking place immediately after the participants’ completion of the program, this reflection may happen 3–4 years later, in their current occupation. The experience gained from the program resembles a seed planted in the heart of the participants and waiting to be nurtured for their career development and personal growth in the future. Indeed, the more senior participants provided more in-depth reflection not just on the outcomes related to personal growth but also environmental awareness and attitude.

This study provides critical insight into the use of retrospective program evaluation to assess the long-term impact of EfS programs. Although this study was not a longitudinal study following the same batch of participants, its cross-sectional examination of different cohorts could serve as a serial time-point sampling approach. Compared with a cross-sectional study for a single cohort, this serial time-point sampling approach can provide more information on the outcomes of an EfS program in the long term. Certainly, the applicability of such an approach depends on whether the EE/EfS program examined was run repeatedly for cohorts. Indeed, many EfS programs are organized in a repetitive manner, which can be assessed by this approach.

### Merits of the Program for Future Reference

The hierarchical organization of the pedagogy of GBL with the sub-pedagogies implemented in the program provides a pedagogically diverse approach to nurture students. The outcomes described by the students for multiple sub-pedagogies imply that the pedagogies might operate in a collective, rather than an idiosyncratic manner. For example, the whole team of students serving in the ecogarden engaged in both collaborative and service-based learning. The students worked together to tackle all of the problems encountered during the management of the garden, gaining learning experience through problem-based, nature-based and collaborative learning. This organization of multiple sub-pedagogies may serve as a multi-pedagogical model or reference for future GBL or other place-related pedagogies.

The non-formal nature of the program exerted no stress on the students. Such stress may interfere with students’ motivation to learn, as indicated in the findings. The 1-year program also gave the students plenty of time to gain experience. The ample time allowed enhanced relationship building among participants, as well as between the teachers and the participants. The close relationships between the members of the whole team (including the teachers) facilitated the overall management of the ecogarden. Indeed, an interviewee commented that she would like to meet the teachers of the program again, years after she had graduated.

Moreover, the training provided by the program in both the cognitive and psychomotor domains, such as knowledge and skills in farming and presentation, facilitated the students’ career development, as suggested by the survey data. The benefits were particularly apparent for pre-service teachers. The alignment of learning content with what is needed for one’s potential career development is another important benefit of this program.

### Limitations

The major limitation of the current study was the relatively small sample involved in the semi-structured interviews. Nevertheless, our findings indicate a relationship between the length of time since completing the program and the number of outcomes described that relate to personal growth, which warrants further comprehensive and vigorous study. The interplay between the experience accumulated in the GBL program and the potential for metacognitive reflection should also be studied further.

## Conclusion

To conclude, the study showed that the annual co-curricular ecogarden-based program in Hong Kong HEIs adopting GBL as the overarching pedagogy with diverse sub-pedagogies could sustain the knowledge and skills learnt by the participants for up to 4 years after the completion of the program. Furthermore, the program aroused environmental awareness and nurtured a positive attitude toward sustainability. The more senior a cohort of participants was, the more in-depth and diversified reflections they had on their personal growth. This result is probably related to the participants’ metacognition of the experience gained in the program during their current occupations. This study provides critical insights into the use of retrospective program evaluation to assess the long-term impact of an EfS program by introducing a cross-sectional study of different cohorts as a serial time-point sampling strategy.

## Data Availability Statement

The raw data supporting the conclusions of this article will be made available by the authors, without undue reservation, to any qualified researcher.

## Ethics Statement

The studies involving human participants were reviewed and approved by Human Research Ethics Committee of the Education University of Hong Kong (Ref. No.: 2019-2020-0312). The patients/participants provided their written informed consent to participate in this study.

## Author Contributions

C-CC, K-HT, and W-CL were the project leaders and contributed to the conception of the study. C-CC was the corresponding author. C-CC, K-HT, and W-CL designed and implemented the co-curricular program with technical and logistical assistance from W-KN. W-KN collected most of the data. W-KN and Y-SW worked with C-CC on data analysis before the writing. All of the authors contributed to the manuscript revision and read and approved the submitted version.

## Conflict of Interest

The authors declare that the research was conducted in the absence of any commercial or financial relationships that could be construed as a potential conflict of interest.

## Supplementary Material

Supplementary Appendix 1 Questions to be asked in online questionnaire.

Supplementary Appendix 2 Questions to be asked in the video conferencing video.
