# Supplementary material for: Planting a Seed of Experience – Long Term Effects of a Co-curricular Ecogarden-Based Program in Higher Education in Hong Kong
Source: Front Psychol. 2021 Jan 15;11:583319. doi: 10.3389/fpsyg.2020.583319 (PMC7843962; doi:10.3389/fpsyg.2020.583319)
Supplement: Supplementary Appendix 1 — Questions to be asked in online questionnaire. [file Table_1.docx]

**Appendix 1.** Questions to be asked in online questionnaire

Personal Information

Name:

Gender :

Programme of study :

Expected Year/ Year of Graduation:

Email:

Contact number:

Occupation (applicable for graduates only) :

Questions for online questionnaire:

1. When did you join our programme? Year ’16/’17, ’17/’18 , ’18/’19 or ’19/’20
2. What is/was your level of participation in the programme?

(Scale 1 to 5; 5: the highest level of participation, 1: the lowest level of participation)

1. On average, how many hours did you spend on the programme in one week?
2. What kind of activities/tasks did you complete in the programme?
3. Why did you join the programme?
4. What did you learn from the programme?
5. How did/do you apply the knowledge and skills acquired from the programme to your daily living and your current career development?
6. Is/was there any subtle change that the programme exerts on you? On which aspect(s)?
7. Which factors make this programme successful/ unsuccessful?
8. What is the most impressive moment for you in joining the programme?
9. Other comments on the programme.
10. Will you be interested and available to conduct a follow-up interview via video conferencing?

**Appendix 2.** Questions to be asked in the video conferencing video

1. Why did you join the programme?
2. What did you expect to learn from the programme before joining it?
3. In the programme, what activities/tasks did you complete?
4. What did you learn from the programme?
5. What was your favourite activity, and why?
6. What were your most memorable experiences in the programme and why?
7. Please describe your memories with other people (such as the staff and other participants) you met in the programme.
8. How did you apply the skills and knowledge acquired in the programme?
9. Please tell me how the experiences of the programme influence you.
10. What meaning, if any, did the programme hold for you?
